# Endoscopic Ultrasound-Guided Hepaticogastrostomy Is Effective for Repeated Recurrent Cholangitis after Surgery: Two Case Reports

**DOI:** 10.1155/2018/7201967

**Published:** 2018-06-10

**Authors:** Akihiro Matsumi, Hironari Kato, Yousuke Saragai, Sho Mizukawa, Saimon Takada, Shinichiro Muro, Daisuke Uchida, Takeshi Tomoda, Kazuyuki Matsumoto, Masaya Iwamuro, Shigeru Horiguchi, Yoshiro Kawahara, Hiroyuki Okada

**Affiliations:** Okayama University Graduate School of Medicine, Dentistry and Pharmaceutical Sciences, Gastroenterology and Hepatology, 2-5-1 Shikata-cho, Kita-ku, Okayama City, Okayama 700-8558, Japan

## Abstract

We report the cases of two patients who underwent endoscopic ultrasound-guided hepaticogastrostomy (EUS-HGS) using metallic stents (MS) for recurrent cholangitis due to benign biliary stenosis. The patients had repeatedly undergone double-balloon endoscopy and anastomotic stenosis. Thus, EUS-HGS was performed. The procedures were successful, and placement of a covered metallic stent (C-MS) relieved cholangitis. The occurrence of cholangitis was subsequently considerably reduced. For patients with postoperative recurrent cholangitis, EUS-HGS with MS should be considered because of its efficacy and safety.

## 1. Introduction

Endoscopic ultrasound-guided biliary drainage (EUS-BD) is now an established technique and is mainly used in patients who fail endoscopic retrograde cholangiopancreatography (ERCP) or in those with reconstructed gastrointestinal anatomy [1]. EUS-BD has been reported in patients with malignant biliary stenosis, but there are few reports in those with benign biliary stenosis [2–5]. We herein describe two cases of endoscopic ultrasound-guided hepaticogastrostomy (EUS-HGS) using metallic stents (MS) for patients with recurrent cholangitis due to benign biliary stenosis.

## 2. Case Reports

### 2.1. Case 1

**Figure 1:**
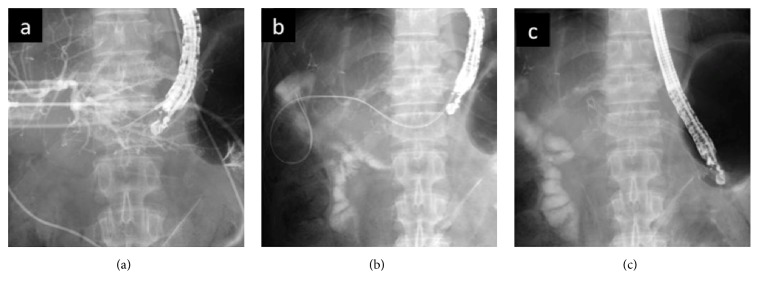
Endoscopic ultrasound-guided hepaticogastrostomy (EUS-HGS). (a) Bile duct is punctured with a 19-G needle. (b) Guidewire is inserted into the bile duct. (c) Metallic stent is placed.

A 58-year-old man underwent pancreatoduodenectomy and right hepatic lobectomy with choledochojejunostomy for a duodenal gastrointestinal stromal tumor with multiple liver metastases. Ten years after the operation, he developed recurrent fever and upper abdominal pain with hepatobiliary enzyme elevation. He underwent double-balloon endoscopy (DBE) and anastomotic stenosis was revealed. There was no evidence of malignancy, and we diagnosed cholangitis due to benign anastomotic stenosis. Balloon dilation for stenosis and biliary stenting with a plastic stent (PS) was performed. As relapsing cholangitis occurred 6 times a year, he underwent EUS-HGS with MS. We used a GF Type UCT 260 (Olympus Medical Systems, Tokyo, Japan) endoscope. The B3 duct was visualized from the stomach. After the absence of blood vessels crossing the puncture route was confirmed, the bile duct was punctured with a 19-G needle (EZ shot 3; Olympus) (Figure 1(a)). Then, a 0.025-inch guidewire (VisiGlide 2; Olympus) was introduced into the jejunum in an antegrade manner. Subsequently, the puncture site was dilated with a 3.6-Fr double-lumen catheter (Uneven Double Lumen Catheter; PIOLAX, Tokyo, Japan), and another 0.035-inch wire (Revowave; PIOLAX, Tokyo, Japan) was introduced into the jejunum (Figure 1(b)). An 8 mm covered MS (Niti-S; TaeWoong Medical Inc., Seoul, Korea) was placed (Figure 1(c)). No adverse events occurred. Before EUS-HGS, fever and hepatobiliary enzyme elevation frequently recurred. After EUS-HGS, the enzymes normalized, and cholangitis has not recurred in 5 months.

### 2.2. Case 2

A 68-year-old man underwent extended right hepatectomy and bile duct resection with choledochojejunostomy for hilar cholangiocarcinoma. Relapsing cholangitis occurred because of anastomotic benign stenosis after the operation. Biliary stenting with PS had repeatedly been performed, but the stenosis did not improve. Thus, he underwent EUS-HGS with MS. The B3 duct was punctured with a 19-G needle (Expect; Boston Scientific, Natick, MA, USA). Then, a 0.025-inch guidewire (Radifocus; Terumo, Tokyo, Japan) was introduced into the jejunum. Subsequently, the puncture site was dilated with a 6-Fr diathermic dilation catheter (Cysto-Gastro-Set; ENDO-FLEX, Voerde, Germany). The wire was changed to another 0.035-inch wire (THSF; Cook Medical, Winston-Salem, NC, USA), and an 8 mm covered MS (Niti-S) was placed. No adverse events occurred. Nine months after EUS-HGS, cholangitis occurred only once due to debris and granulation. We performed balloon sweeping for debris and placed a PS into the MS. Twelve months after EUS-HGS, we replaced the PS with an MS. As in case 1, hepatobiliary enzymes normalized, and cholangitis has not recurred in 11 months.

## 3. Discussion

Postoperative benign biliary stenosis at the anastomotic site is one of the most common complications [6–8]. Reoperation or dilation of stenosis through the percutaneous transhepatic biliary drainage route has usually been performed, but these are invasive and negatively influence the quality of life. Endoscopic therapy is less invasive and has recently been indicated for benign biliary stenosis. However, this is problematic in patients with surgically altered anatomy because of difficulty advancing the endoscope to the anastomotic site, especially after right lobectomy (Figure 2①). Although balloon enteroscopy has enabled endoscopic therapy for these patients and balloon dilation and/or biliary stenting can be performed, repeated treatments are often needed due to relapsing cholangitis.

**Figure 3:**
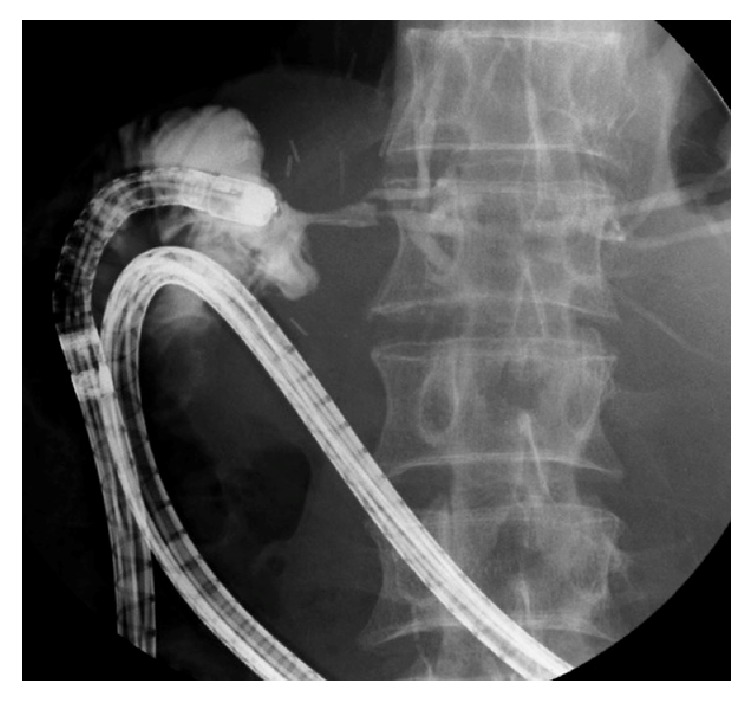
Contrast agent was stagnant around the anastomosis.

In these two cases, repeated drainage with plastic stents was performed; nevertheless, relapsing cholangitis occurred too soon afterward to be explained by stent obstruction. We think that relapsing cholangitis occurred because of an anatomical problem in the afferent loop. When we performed biliary stenting with DBE in Case 2, contrast agent was stagnant around the anastomotic area (Figure 3). The stagnation of contrast agent was due to a deformity of the afferent loop caused by adhesions and/or a long afferent loop, which leads to stagnation of bile around the choledochojejunal anastomosis and recurrent cholangitis (Figure 2②). A pathway for bile other than through the afferent loop can avoid or reduce stagnation. Therefore, we think that EUS-HGS with MS can reduce recurrences of cholangitis and achieve long-term patency.

EUS-BD has become accepted therapy for patients who fail conventional ERCP [9, 10]. Although many cases have been reported in patients with malignant stenosis, few studies have reported results with benign stenosis. Prospective studies with long-term follow-up are needed to confirm the feasibility and safety of EUS-HGS with MS for benign postoperative stenosis.

## Figures and Tables

**Figure 2:**
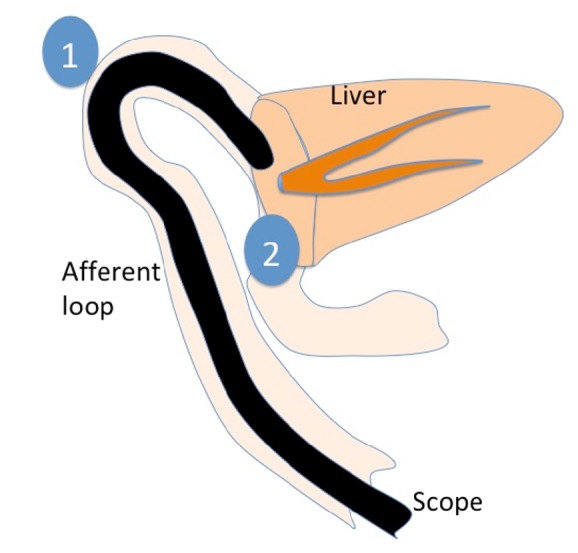
Because the afferent loop invaginates into the space after right lobectomy, the scope is difficult to move on (①) and bile juice is easy to stagnate (②).

## References

[1] Sharaiha R. Z., Khan M. A., Kamal F. (2017). Efficacy and safety of EUS-guided biliary drainage in comparison with percutaneous biliary drainage when ERCP fails: a systematic review and meta-analysis. *Gastrointestinal Endoscopy*.

[2] Katanuma A., Maguchi H., Osanai M., Takahashi K. (2012). Endoscopic ultrasound-guided biliary drainage performed for refractory bile duct stenosis due to chronic pancreatitis: A case report. *Digestive Endoscopy*.

[3] Moole H., Bechtold M. L., Forcione D., Puli S. R. (2017). A meta-analysis and systematic review: Success of endoscopic ultrasound guided biliary stenting in patients with inoperable malignant biliary strictures and a failed ERCP. *Medicine*.

[4] Khan M. A., Akbar A., Baron T. H. (2016). Endoscopic Ultrasound-Guided Biliary Drainage: A Systematic Review and Meta-Analysis. *Digestive Diseases and Sciences*.

[5] Khashab M., Van der Merwe S., Kunda R. (2016). Prospective international multicenter study on endoscopic ultrasound-guided biliary drainage for patients with malignant distal biliary obstruction after failed endoscopic retrograde cholangiopancreatography. *Endoscopy International Open*.

[6] Goldman L. D., Steer M. L., Silen W. (1983). Recurrent cholangitis after biliary surgery. *The American Journal of Surgery*.

[7] Ammori B. J., Joseph S., Attia M., Lodge J. P. A. (2000). Biliary strictures complicating pancreaticoduodenectomy. *International Journal of Gastrointestinal Cancer*.

[8] Shiihara M., Miura O., Konishi K. (2016). A case of postoperative recurrent cholangitis after pancreaticoduodenectomy successfully treated by tract conversion surgery. *Journal of Surgical Case Reports*.

[9] Siripun A. (2015). Endoscopic ultrasound-guided biliary intervention in patients with surgically altered anatomy. *World Journal of Gastrointestinal Endoscopy*.

[10] Khashab M., El Zein M., Sharzehi K. (2016). EUS-guided biliary drainage or enteroscopy-assisted ERCP in patients with surgical anatomy and biliary obstruction: an international comparative study. *Endoscopy International Open*.

